# Angiogenic Factor-Based Signature Predicts Prognosis and Immunotherapy Response in Non-Small-Cell Lung Cancer

**DOI:** 10.3389/fgene.2022.894024

**Published:** 2022-05-18

**Authors:** Xinpei Gu, Liuxi Chu, Yanlan Kang

**Affiliations:** ^1^ Department of Human Anatomy, Shandong First Medical University and Shandong Academy of Medical Sciences, Taian, China; ^2^ School of Biological Sciences and Medical Engineering, Southeast University, Nanjing, China; ^3^ Institute of AI and Robotics, Academy for Engineering and Technology, Fudan University, Shanghai, China

**Keywords:** NSCLC, angiogenic factors, immunotherapy response, model validation, biomarkers

## Abstract

Non-small-cell lung cancer (NSCLC) is one of the most common malignancies, and specific molecular targets are still lacking. Angiogenesis plays a central regulatory role in the growth and metastasis of malignant tumors and angiogenic factors (AFs) are involved. Although there are many studies comparing AFs and cancer, a prognostic risk model for AFs and cancer in humans has not been reported in the literature. This study aimed to identify the key AFs closely related to the process of NSCLC development, and four genes have been found, C1QTNF6, SLC2A1, PTX3, and FSTL3. Then, we constructed a novel prognostic risk model based on these four genes in non-small-cell lung cancer (NSCLC) and fully analyzed the relationship with clinical features, immune infiltration, genomes, and predictors. This model had good discrimination and calibration and will perform well in predicting the prognosis of treatment in clinical practice.

## 1 Introduction

Lung cancer is one of the malignant tumors with the highest incidence and mortality worldwide (Hirsch et al., 2017). Every year, 1.8 million people (11.6% of total cases) are diagnosed with lung cancer, and about 1.6 million people (18.4% of total cancer deaths) died because of lung cancer. There are two basic forms of lung cancer, small cell lung cancer (SCLC) and non-small-cell lung cancer (NSCLC), and NSCLC accounts for approximately 85% (Ettinger et al., 2013; Gridelli et al., 2015). NSCLC is characterized by poor survival, and despite significant advances in new chemotherapeutic drugs and clinical surgery, the prognosis remains suboptimal (Ettinger et al., 2013; Gridelli et al., 2015; Ettinger et al., 2021). With the advent of targeted molecular therapy and immune checkpoint inhibitors, the use of biomarkers in identifying patients is becoming increasingly common (Ma et al., 2019; Wang et al., 2019). The existing evidence has suggested that targeted therapies have favorable therapeutic effects. However, acquired resistance has become a major obstacle in the field of targeted therapies (Chatterjee and Bivona 2019). Thus, more novel driver genes, therapeutic targets, and prognostic biomarkers must be discovered and used for targeted therapy in larger populations, more accurate prognosis prediction, and a better understanding of the mechanisms of lung cancer development.

Tumors can promote tumor angiogenesis, leading to angiogenesis, which is the one of hallmarks of cancer (Hanahan and Weinberg 2000). The process of new blood vessel formation is critical in supporting tumor growth, and solid tumors secrete angiogenic factors (AFs) implicated in the complex regulation of angiogenesis (Goveia et al., 2020). Numerous important target molecules of AFs in NSCLC and other cancers, such as vascular endothelial growth factor (VEGF) (Zhang 2015) and epidermal growth factor receptor (EGFR) (Oxnard et al., 2011), have all become clinical targets for antitumor angiogenesis. Antiangiogenic medications are increasingly used as anticancer drugs for first-line treatment. Moreover, since the introduction of the first humanized anti-VEGF monoclonal antibody, bevacizumab (Avastin), available in 2004 (Ferrara et al., 2005), there have been nearly 30 antiangiogenic drugs approved by the FDA (Lugano et al., 2020). AFs are also expected to be optimal therapeutic targets. Several significant global studies noted that angiogenesis inhibitors combined with immunotherapy can enhance the curative effect. There is increasing evidence that targeting angiogenesis improves the efficiency of cancer immunotherapy. A programmed cell death 1 (PD-1) inhibitor and camrelizumab (AiRuiKa™) can improve the treatment effect of chemotherapeutics in multiple types of cancers (Markham and Keam 2019). However, apatinib, a vascular endothelial growth factor receptor 2 (VEGFR2) tyrosine kinase inhibitor, has been shown to increase the infiltration of CD8^+^ T cells, reduce the recruitment of tumor-associated macrophages, and improve the effect of PD-1 inhibitors (Zhao et al., 2019).

Despite many studies investigating the association between AFs and cancers, whether AFs can be used as biomarkers to predict the prognosis of NSCLC patients is still unknown. In our study, based on the machine algorithms and bioinformatics methods, AF-related risk score (AFRS) was established. Four key prognosis-related AFs, C1QTNF6, SLC2A1, PTX3, and FSTL3, were first screened using bioinformatics analysis of differentially expressed genes (DEGs). Then, we attempted to construct a new risk score model to predict NSCLC, and we further analyzed the clinical features, immune infiltration, genomes, and multiple predictors. To further validate the AF-related prognostic risk score model, we used external dataset validation. An overview of this study is shown in Supplementary Figure S1.

## 2 Results

The expression profile data of NSCLC patients were downloaded from the UCSC database. The detailed clinical features of these patients are summarized in Table 1.

**TABLE 1:** Clinic pathological data of patients with NSCLC in this study.

Characteristic		Number
Age	<60	720
	≥60	221
Pathologic_M	M0	698
	M1	30
	MX	208
	NA	5
Pathologic_N	N0	600
	N1	213
	N2	106
	N3	7
	NX	14
	NA	1
Pathologic_T	T1	262
	T2	529
	T3	108
	T4	39
	TX	3
Clinical stage	Ⅰ	476
	Ⅱ	264
	Ⅲ	159
	Ⅳ	31
	NA	11
Follow up status	Alive	570
	Dead	371

### 2.1 Differential Expression Analysis and Functional Enrichment Analysis of Non-Small-Cell Lung Cancer

**FIGURE 1:**
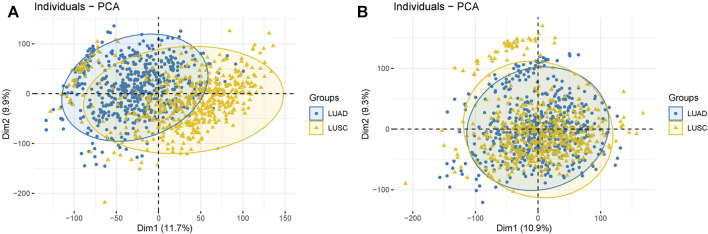
Batch effect removal. **(A)** Before batch effects were removed. **(B)** After batch effects were removed.

We identified a total of 372 differentially expressed AF genes in cancer and normal samples (with a threshold of adj.P.Val<0.01 & |log (FC) |≥1) (Figures 1A,B). GO and KEGG functional enrichment analyses of the differentially expressed AF genes were then performed (Figures 1C–F). The enriched GO terms of DEGs were classified into three categories: molecular functions, cellular components, and biological processes. The results revealed that these genes were enriched for GO terms such as regulation of vasculature development, regulation of angiogenesis, ameboidal-type cell migration, and positive regulation of vasculature development, epithelial cell proliferation, and tissue migration. The KEGG pathway enrichment showed the enrichment of critical pathways involved in tumorigenesis and metastasis, including pathways in cancer, focal adhesion, the MAPK signaling pathway, the chemokine signaling pathway, the TGF-β signaling pathway, and renal cell carcinoma. The top 15 highly enriched KEGG pathways are presented in Figure 1F.

### 2.2 Cox Regression Analysis of Differentially Expressed Angiogenic Factor Genes

**FIGURE 2:**
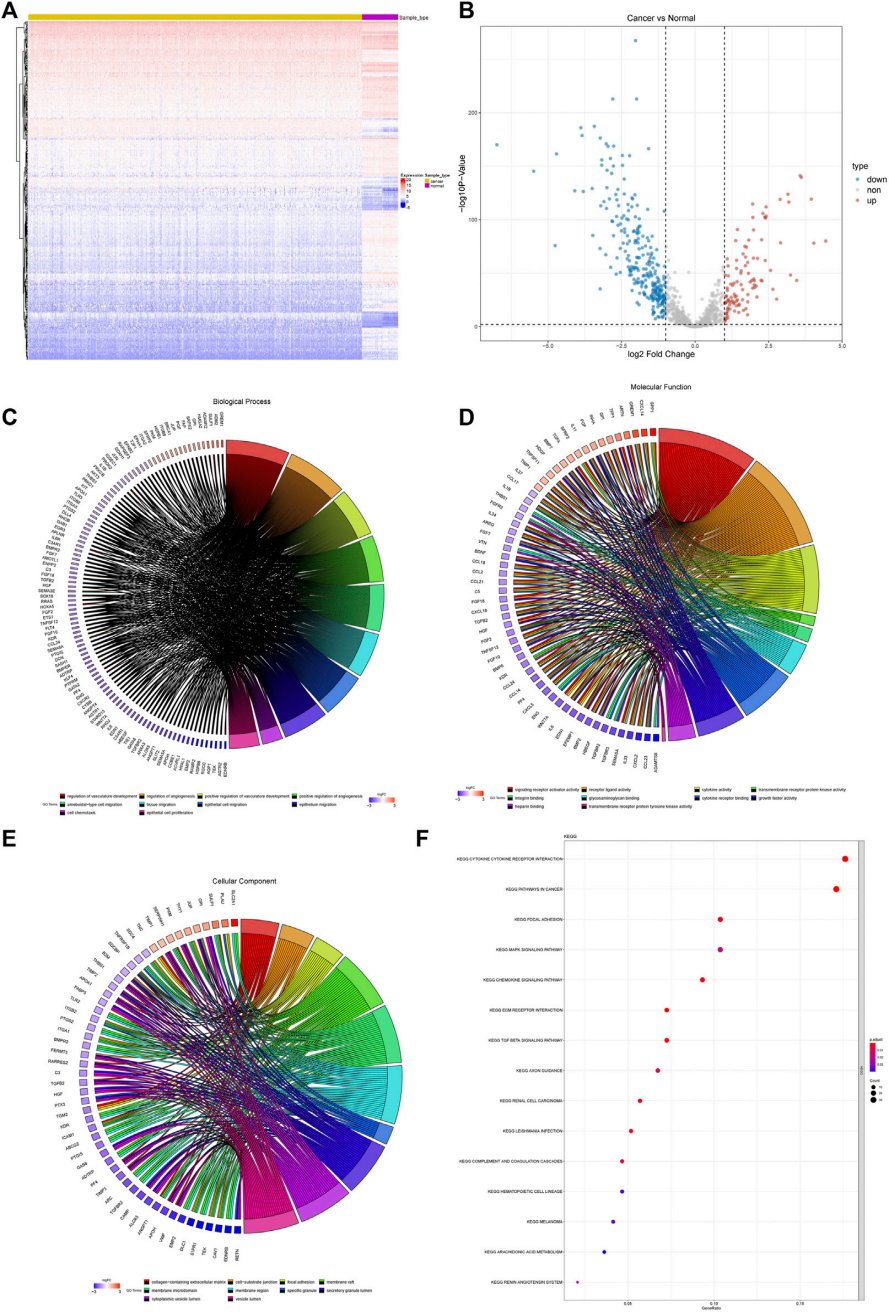
Differential expression and functional enrichment of AF genes in non-small-cell lung cancer. **(A)** Heatmap and clustering of differentially expressed AF genes. **(B)** Volcano map of differentially expressed AF genes. **(C)** GO biological processes **(D)** GO molecular functions **(E)** GO cellular components and **(F)** KEGG.

We performed a univariate Cox regression analysis of these differentially expressed AF genes and identified 58 AF-related genes that were associated with the prognosis of NSCLC. We performed survival analyses of the top five genes in terms of the *p*-value (Figures 2A–E). The low expression of these five genes was associated with a worse prognosis (Figure 2F).

### 2.3 Development of Risk Model Using Lasso Regression

A total of five AF genes significantly associated with prognosis (*p* < 0.001) in the univariate Cox regression were further selected for lasso regression (Supplementary Figure S2). We first used cross-validation to identify the minimal lambda, i.e. lambda min, and then selected the four most significant genes using lambda min to develop the prognostic risk model. The optimized model was: risk score = 0.104 * SLC2A1 + 0.138 * FSTL3 + 0.069 *C1QTNF6 + 0.046 * PTX3. We calculated the risk scores of each sample using this formula and classified all the samples into high- and low-risk groups according to the median for further analysis.

**FIGURE 3:**
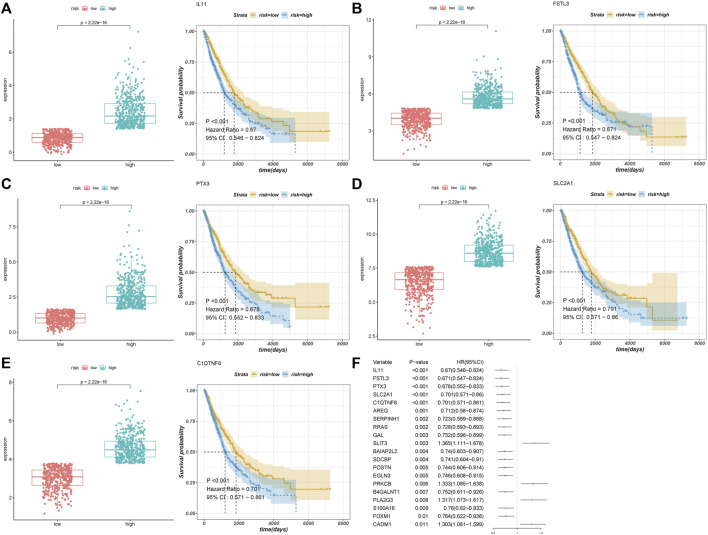
Univariate Cox regression analysis. **(A–E)** Top five prognostic genes. **(F)** Forest plot of the top 20 genes.

To validate the performance of our model, we plotted the Kaplan–Meier survival curves of the high- and low-risk groups (Figure 3A). A significant association was shown between the risk group and survival (*p* < 0.0001), suggesting that the model had a high prognostic value. Time-dependent ROC curves were further plotted, which showed AUC>0.6 in the 1-year, 3-years, and 5-years ROC curves. This indicated that the model had good prediction ability (Figure 3B). Based on the optimistic cutoff, the patients were divided into high AF risk score and low AF risk score groups (Figures 3C–F).

**FIGURE 4:**
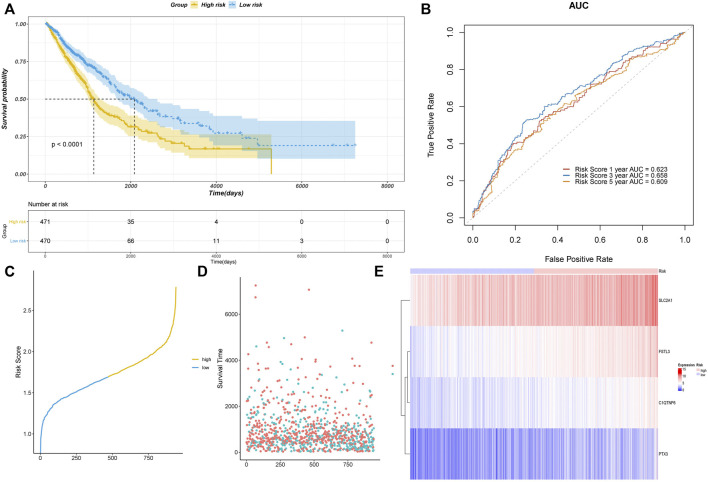
Assessment of the risk model based on TCGA data. **(A)** Kaplan–Meier curve validation. **(B)** ROC curve validation. **(C)** Risk score of all samples. **(D)** Scatter plot of the survival time of all samples. **(E)** Heatmap of the prognostic genes in high- and low-risk groups.

We used the GSE4573 and GSE68465 datasets to validate the model (Figures 4A–D). We combined the two datasets and removed the batch effect. We selected the prognostic genes in the datasets (C1QTNF6 was not identified) and calculated the risk score using the coefficients in the model for validation. The Kaplan–Meier plot showed that the samples in the high-risk group had a worse prognosis with a *p*-value < 0.05, which indicated that our model had high accuracy.

### 2.4 Differential Analysis and Association Analysis of the Angiogenic Factor Risk Score

**FIGURE 5:**
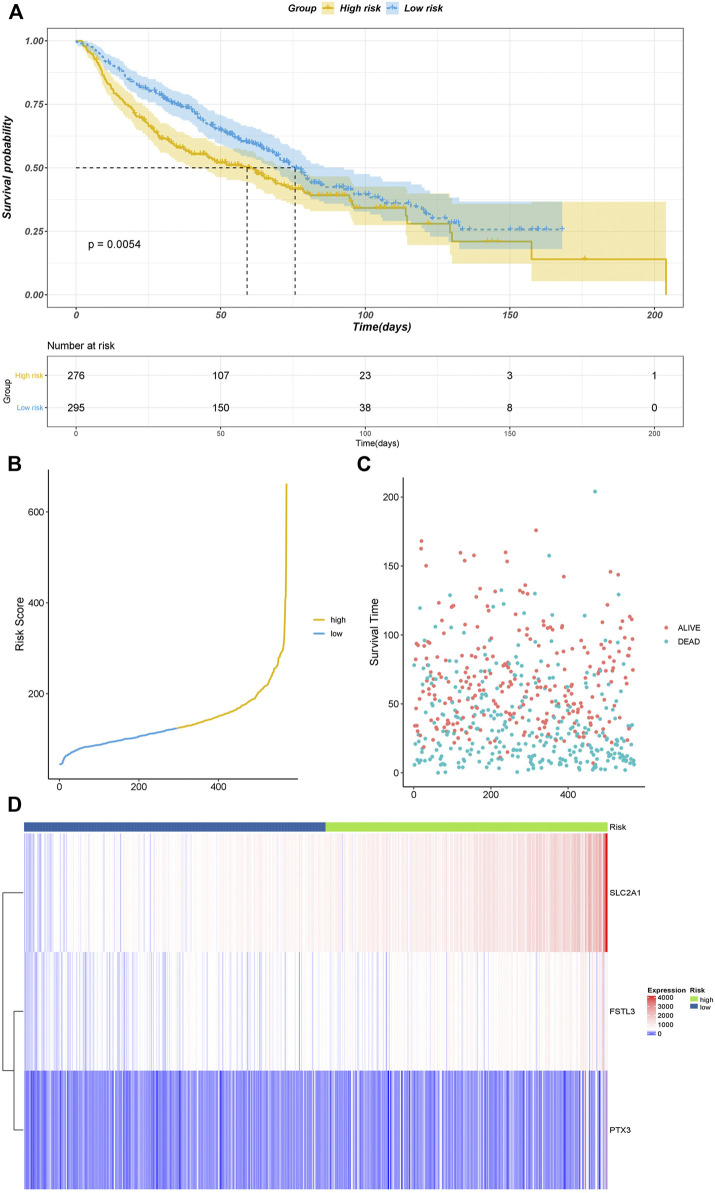
Validation results of datasets GSE4573 and GSE68465. **(A)** Kaplan–Meier plot. **(B)** Risk score of all samples. **(C)** Scatter plot of the survival time of all samples. **(D)** Heatmap of the prognostic genes in high- and low-risk groups.

We analyzed the difference in AF risk scores of each group that was stratified by clinical characteristics. The risk score of LUSC was significantly higher than that of LUAD (Figure 5A). The risk score of the samples with EGFR mutations was significantly lower than that of samples without EGFR mutations (Figure 5B). The risk score also differed significantly across the different tumor stages and TNM stages, which was consistent with the process of carcinogenesis (Figures 5C–F). The patients with a smoking history also had significantly higher risk scores than those who never smoked (Figure 5G).

**FIGURE 6:**
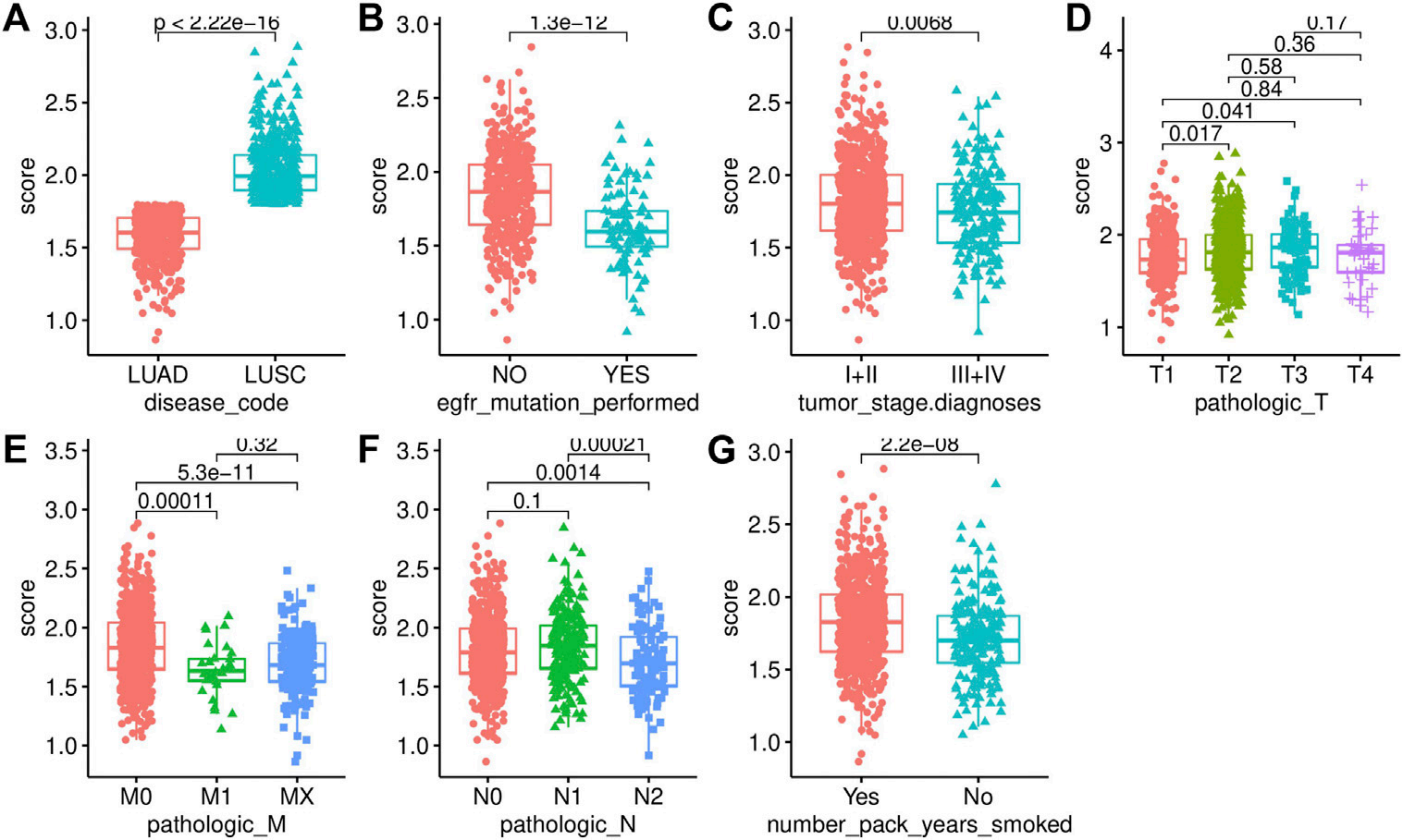
Association analysis with clinical characteristics. **(A)** Disease code. **(B)** EGFR mutation status. **(C)** Tumor stage. **(D)** T stage. **(E)** M stage. **(F)** N stage. **(G)** Smoking history.

We also visualized the association of the risk score with tumor mutational burden (TMB), homologous recombination deficiency (HRD), neoantigen burden, chromosomal instability (CIN), and stemness index (mRNAsi) (Figures 6A–D). TMB is a marker for genomic instability measured by sequencing the whole tumor genome and has been shown to correlate with immunotherapy (Gibney et al., 2016). Therefore, TMB is emerging as a predictor of immunotherapeutic responses. For all indexes, the highest correlation was obtained for TMB (Figure 6A). This further illustrates that the interaction of AFs can affect immunotherapy. The discovery of homologous recombination deficiency (HRD) in lung cancer is of great importance for patients who will benefit from poly ADP-ribose polymerase inhibitor (PARPi) (Weston et al., 2010). However, we did not find a correlation between HRD and AFs (Figure 2B). Neoantigens are another important index for predicting the clinical response to immunotherapy. The current studies of neoantigen sources mainly focus on single nucleotide variants (SNVs), such as small insertions and deletions (indels), somatic copy number variations (SCNVs), and large scale transition (LSTms). Similarly, we found no significant differences in these parameters (Figures 6C–H). The stemness index (mRNAsi) is used to measure the tumor development and evaluate the reliability of stem cell indexes as shown in Figures 6A,I significant positive correlation was found between AFs and mRNAsi. These results confirmed that AFs were related to biological processes, cancer metastasis, and the immune microenvironment.

### 2.5 Immune Infiltration Analysis of High- and Low-Risk Groups

**FIGURE 7:**
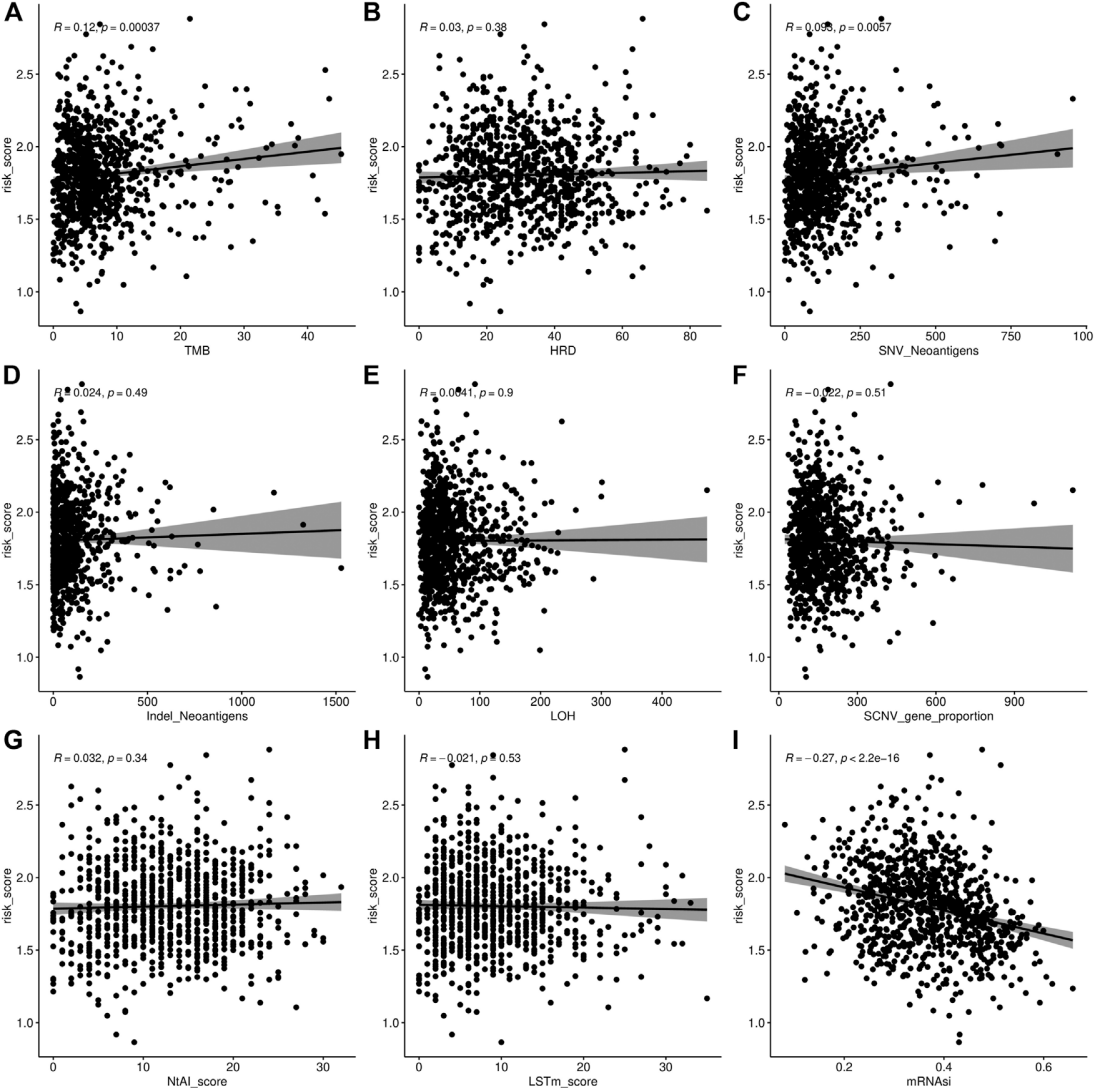
Association analysis of AF risk score. **(A)** Tumor mutational burden and AF risk score. **(B)** Homologous recombination deficiency and AF risk score. **(C–D)** Neoantigen burden and AF risk score. **(E)** Loss of heterozygosity (LOH) in chromosome instability and AF risk score. **(F)** SCNV of chromosome instability and AF risk score. **(G)** Telomeric allelic imbalance (NtAI) of chromosome instability and AF risk score. **(H)** Large scale transition (LSTm) **(I)** Stemness index and AF risk score.

The immune infiltration status was highly associated with the prognosis of NSCLC. We used the CIBERSORT algorithm to calculate and compare the proportion of immune infiltration in the high- and low-risk groups based on TCGA data (Figure 7A). The proportions of naive B cells, memory activated CD4 T cells, gammadelta T cells, and resting dendritic cells were significantly increased in the low-risk group, while the proportions of memory B cells, and macrophages. M0, macrophages. M2, and activated mast cells was significantly higher in the high-risk group, which indicated that the immune infiltration status was different in the high- and low-risk groups.

We also found that the stroma score (*p* = 7.8e-16), immune score (*p* = 0.012), and tumor purity (*p* = 1.7e-08) were significantly higher in the high-risk group than in the low-risk group (Figures 7B–D).

### 2.6 Differences in the Mutation Profile Between High- and Low-Risk Groups

**FIGURE 8:**
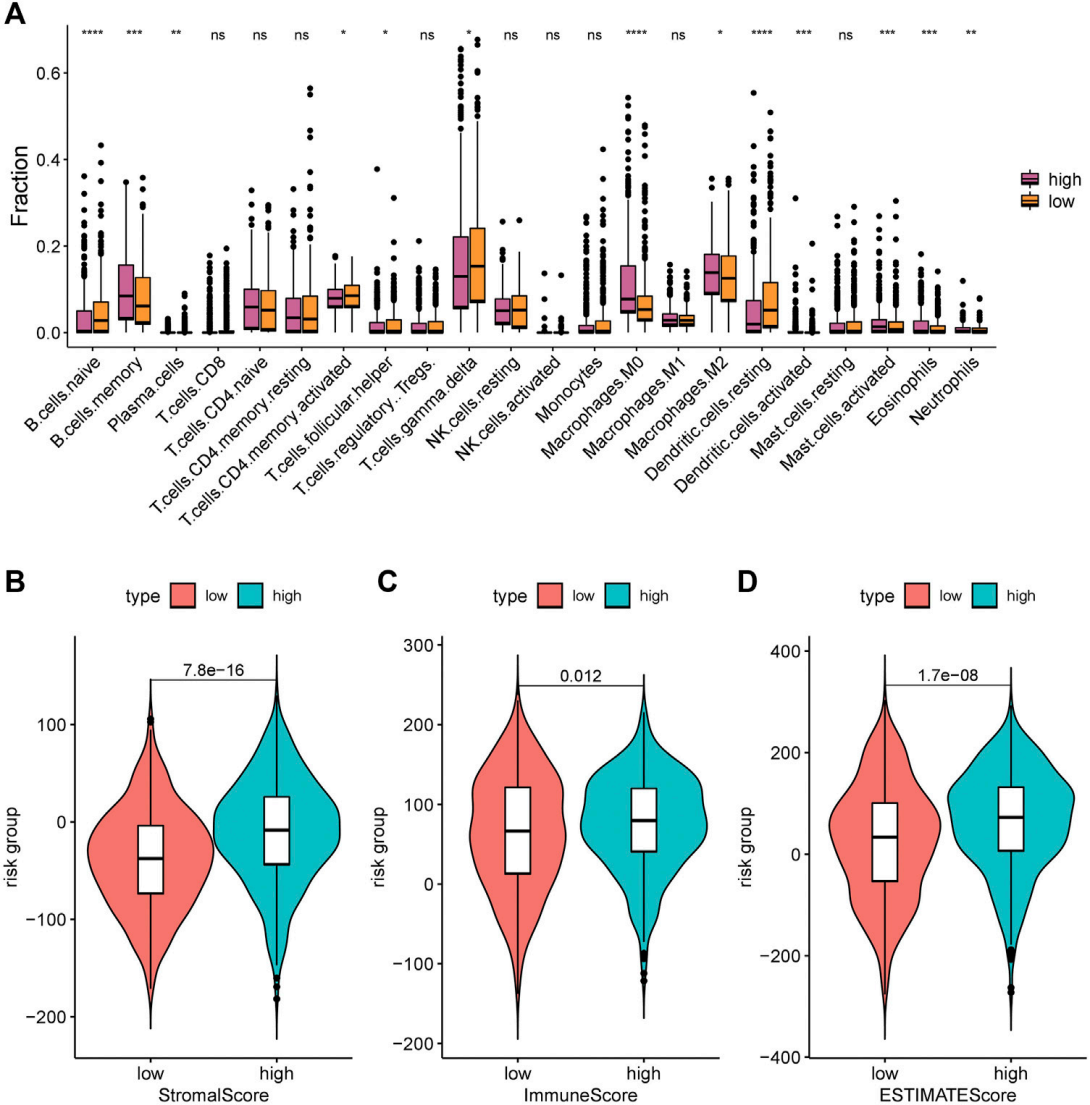
Immune infiltration levels of 22 immune cell types in the low-risk group and high-risk group. **(A)** CIBERSORT algorithm was used to assess the difference in immune infiltration: *, *p* < 0.05; **, *p* < 0.001; ***, *p* < 0.01; ****, *p* < 0.001; ns, *p* > 0.05 (nonsignificant). **(B)** Stromal score; **(C)** Immune score; and **(D)** ESTIMATE score.

We further investigated the difference in the mutation profiles between the high- and low-risk groups based on TCGA data. The mutation rate of the high-risk group was slightly higher than that of the low-risk group (92.81 vs. 90.91%). The mutation rate of TP53 was the highest in both the high- and low-risk groups. Additionally, missense mutations were the most dominant among all mutation types. Single nucleotide polymorphisms (SNPs) occurred more frequently in the high-risk group than in the low-risk group (Figures 8A,B).

We also investigated the difference in CNV between the high- and low-risk groups (Figures 8C–E). The copy numbers of amplification and deletion were distributed differently in the same position. Significant differences in distribution could be observed in the figures (Figures 8C,D). We analyzed the *Z*-score of the high- and low-risk groups (Figure 8E) by *t*-test. The results showed a significant difference between them (*p* < 2.22e-16).

### 2.7 Independent Prognosticator Analysis of Risk Score

**FIGURE 9:**
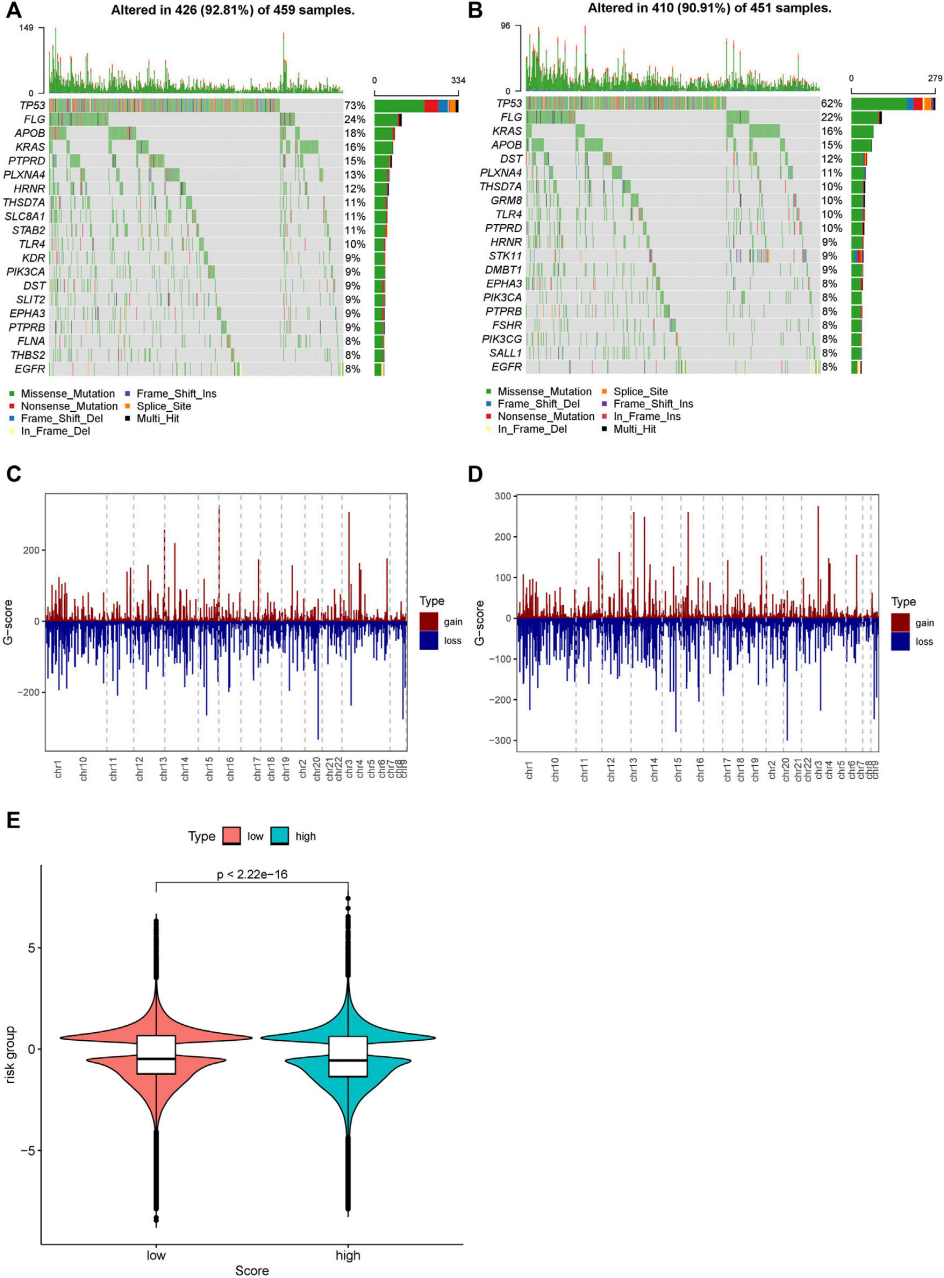
Distribution of mutations and CNVs in the high- and low-risk groups. **(A)** Mutations in the high-risk group. **(B)** Mutations in the low-risk group. **(C)** CNVs in the low-risk group. **(D)** CNVs in the high-risk group. **(E)** Distribution of the G-score and the *p*-value of the Wilcoxon test in the high- and low-risk groups.

Immunotherapy offers a new approach to cancer treatment. For a long period of time, immunotherapy approaches targeting PD1, PDL1, and ctla-4 have all been successfully applied in cancer, with largely promising outcomes. Tumor immune dysfunction and exclusion (TIDE) is a gene expression biomarker developed for predicting the clinical response to immune checkpoint blockade. We used the TIDE score to assess the performance of the risk score to predict the response to immunotherapy and visualized it in R software. A significant difference in the TIDE score was demonstrated between the high- and low-risk groups (*p* = 0.0027) (Figure 9A), while its prediction accuracy was lower than that of the risk score (Figure 9B).

**FIGURE 10:**
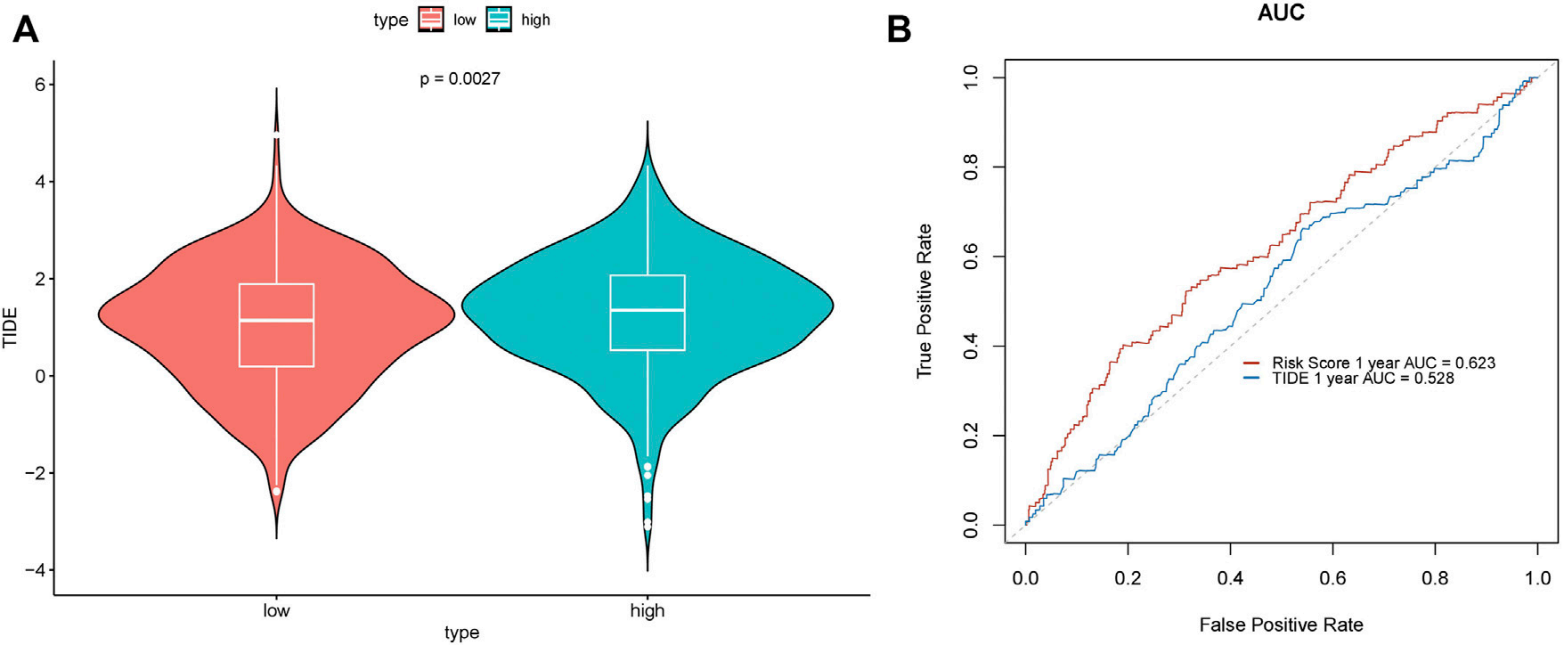
Prediction performance of the TIDE score. **(A)** Difference in TIDE scores in the high- and low-risk groups. **(B)** ROC curve.

To assess the effect of the risk score on prognosis, we performed univariate and multivariate Cox regression analyses of the above clinical characteristics and validated the risk model using validation datasets (Figures 10A–D). The risk score showed a significant effect on the prognosis in both univariate and multivariate regression analyses.

### 2.8 Prognostic Analysis of Risk Score and Clinical Characteristics

**FIGURE 11:**
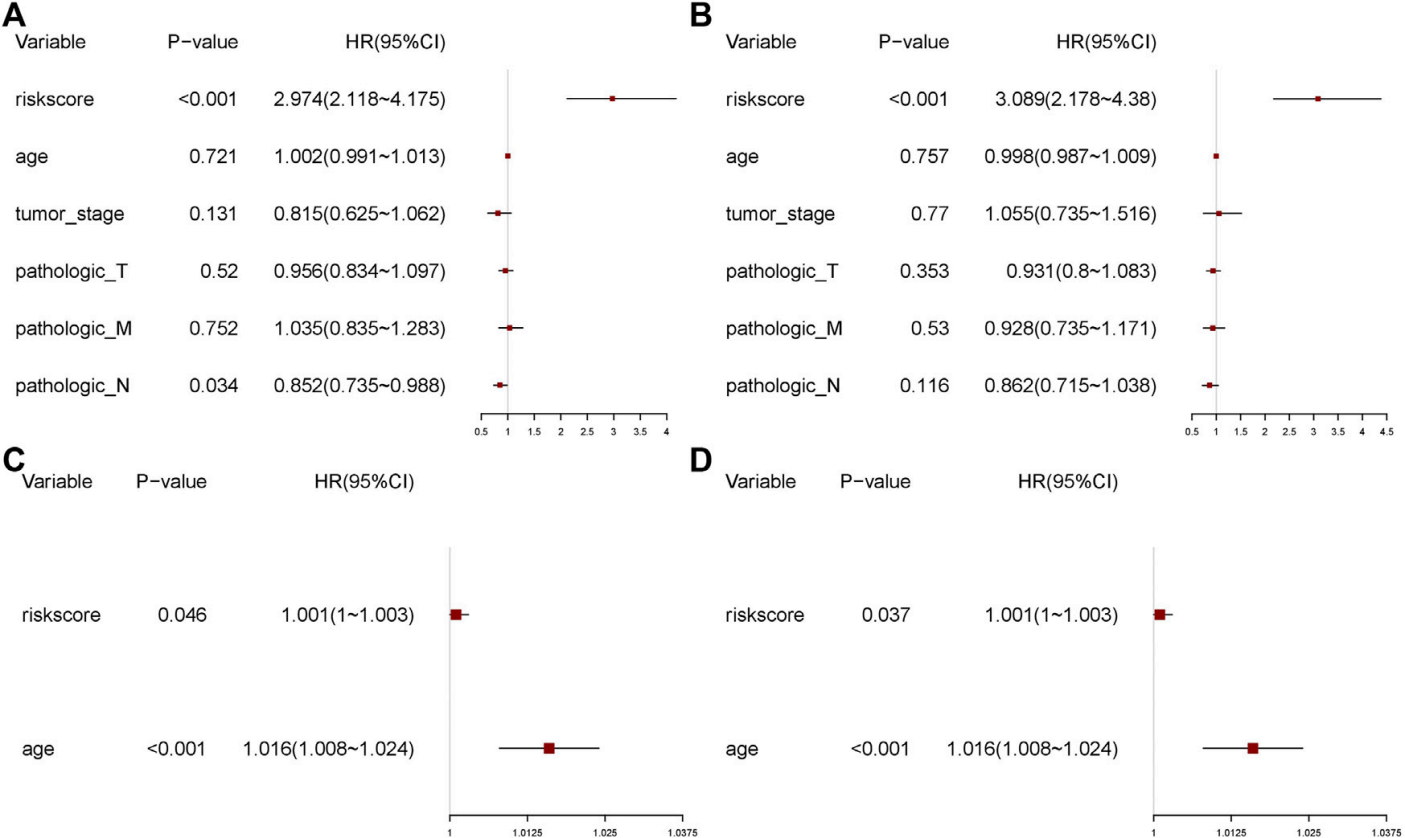
Univariate and multivariate regression analyses. **(A)** Univariate analysis. **(B)** Multivariate analysis. **(C)** Univariate analysis of the validation set. **(D)** Multivariate analysis of the validation set.

Finally, we developed nomograms using the risk score and clinical characteristics and validated them with calibration plots (Figure 11A). The risk score showed the highest accuracy of prediction (Figure 11B). The 1-year, 2-years, and 3-years calibration plots demonstrated the highest accuracy of our nomograms (Figures 11C–E).

## 3 Discussion

Angiogenesis is essential for tumor growth and metastasis and can provide space and nutrients for tumor cells. Multiple angiogenic growth factors play critical roles in this process. The previous studies indicate that targeting tumor angiogenesis is a promising way to fight tumor growth and dissemination in numerous types of cancer (DeBusk et al., 2010; Meng et al., 2017; Chu et al., 2021; Pan et al., 2021).

With the development of next-generation sequencing, more extensive molecules have been discovered as therapeutic targets. However, no study has previously constructed a prediction model of NSCLC based on AFs. In this study, we first identified 372 DE-AFs based on the UCSC database and then confirmed that four genes, C1QTNF6, SLC2A1, PTX3, and FSTL3, were significantly correlated with prognosis by constructing Cox regression and Lasso regression models. High expression of the four genes was also associated with poor prognosis in NSCLC patients. Second, according to the medium-risk score, NSCLC patients were divided into high- and low-risk groups. We calculated each AUC value of the ROC curves for predicting prognosis, which all had significantly good sensitivity. The 1-, 3-, and 5-years AUC values of the ROC were 0.623, 0.658, and 0.609, respectively. The risk score also performed well in validation sets GSE4573 and GSE68465. We also evaluated our AF risk score models on GSE4573 and GSE68465 validation data (batch effect correction). The results showed significant differences between the high- and low-risk groups.

The results of our study were consistent with those of other past studies. Wei et al. (Zhang and Feng 2021) reported that C1QTNF6 was significantly highly expressed in NSCLC tissues and cells and regulated tumor growth, migration, and apoptosis. Similar results have been reported in Japan (Tamotsu et al.) (Takeuchi et al., 2011), in which C1QTNF6 has been implicated in tumor angiogenesis in hepatocellular carcinoma. Solute carrier family 2 member 1 protein (SLC2A1) plays an important role in glucose metabolism in the human body. A previous study suggested that the upregulated expression level of SLC2A1 may increase the tumor cell proliferation and metastasis (Xiong et al., 2020). Hongwei et al. (Xia et al., 2021) found that lncRNA PVT1 can regulate cell growth, migration, and invasion by targeting the miR-378c/SLC2A1 axis. PTX3 is involved in tumor progression in multiple types of cancer and has also been identified as an independent prognostic predictor of cancer (Bonavita et al., 2015; Giacomini et al., 2018). Follistatin-related gene 3 (FSTL3) was proven to be an oncogene, and upregulated the expression of FSTL3 could activate migration by promoting F-actin and BMP/SMAD signaling (Chu et al., 2020; Liu et al., 2021). Although C1QTNF6, SLC2A1, PTX3, and FSTL3 may serve as potential targets for antiangiogenic therapeutic strategies, the molecular mechanisms of angiogenesis remain unclear.

Third, to better guide clinical decision-making, we applied AFRS to different clinical samples. We were pleasantly surprised that AFRS in LUSC patients was significantly higher than that in LUAD patients. AFRS was significantly lower in the patients with EGFR mutation or without smoking. Furthermore, we conducted a correlation between AFRS and different clinical stages and found that AFRS was closely related to the clinical stage and TNM stage.

Fourth, in recent research, immunotherapy has been increasingly recognized for its potential therapeutic effect on a variety of tumors. For example, immune checkpoint (PD-1, CTLA-4) blockade has become an increasingly important part of cancer therapy (Passiglia et al., 2021). There were plenty of clinical trials (Reck et al., 2019; Herbst et al., 2020; Patel et al., 2020) that proved the combination of ICI therapy and angiogenesis therapy can reprogram the immune microenvironment and prune cancer growth-related blood vessels (Ramjiawan et al., 2017; Yi et al., 2019; Giannone et al., 2020), which could have a synergistically better performance in prolonging overall survival, especially in patients with activating mutations of EGFR (Reck et al., 2019). By detecting the immunity indexes of TMB and mRNAsi, we believed that this research might provide bioinformatics evidence to support the design of a combination of immunotherapy and antiangiogenic therapy for NSCLC patients in the future.

Fifth, we found that of all clinical samples, the TP53 mutation type had the highest rate of mutations, neither in the low nor high AFRS group. The SNP mutation in the high AFRS group was remarkably higher than that in the low AFRS group. Numerous studies have proven that TP53 mutation in cancers can influence drug activity, tumor apoptosis, and immune evasion (Alexandrova et al., 2015; Srihari et al., 2018). Notably, gain-of-function p53 mutation promotes neutrophils to tumors, which leads to resistance to immunotherapy (Siolas et al., 2021). As a result, we further analyzed the correlation of AFRS with the infiltration of various immune cells. We found that the immune response was significantly altered between the low and high AFRS groups, including immune cell infiltration (i.e., M2 macrophages and M0, mast cells, B cells), immune score, stromal score, and ESTIMATE score. These results indicated that the high AFRS group could induce stronger immunity activity.

For better clinical applications, we strive to develop a nomogram to predict the prognosis of NSCLC patients. The established nomogram showed a great performance in predicting the clinical outcomes for NSCLC patients.

However, this study has several limitations. First, due to limited resources and funding available, no clinical samples were analyzed, hence, clinical relevance was not assessed. Second, the lack of experimental verification was also limited. We will further confirm our conclusions by performing cell line and animal model experiments in the future and prove the changes in the protein levels by western blot analysis.

## 4 Conclusion

In conclusion, assessing the global gene expression profile of Afs in this study was the first. From the perspective of a reliable risk score model using angiogenic factors, the present study provided a new method for NSCLC treatment in the clinic. However, the established model needs to be further confirmed in the future by large scale multicenter clinical studies.

## 5 Materials and Methods

### 5.1 Sources of Non-Small-Cell Lung Cancer Datasets

**FIGURE 12:**
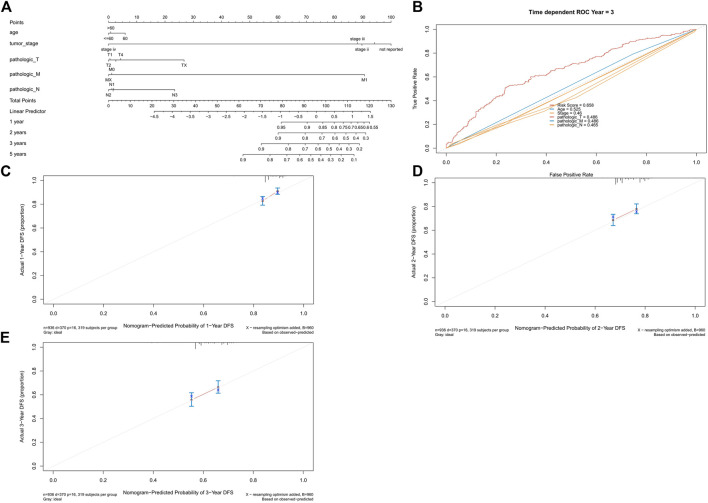
Nomogram and calibration plots. **(A)** Nomogram of age, tumor stage, and TNM stage. **(B)** ROC curve of risk score, age, tumor stage, and TNM stage. **(C)** 1-year calibration plot. **(D)** 2-years calibration plot and **(E)** 3-years calibration plot.

The expression profile combined with patients’ clinical and annotation information in LUAD and LUSC datasets were downloaded from UCSC (https://xenabrowser.net/). Next, we averaged the expression level of genes with the same name and removed the genes with expression levels lower than 30%. We merged the two expression profiles after processing and converted the data type from FPKM to TPM. The samples from patients aged >18 years were extracted, and batch effects were removed (Figures 12A,B). We then searched the NCBI database (https://www.ncbi.nlm.nih.gov/gene/?term=angiogenic) using “angiogenic factor (AF)” as the keyword and extracted AF expression data of 1,054 samples from the downloaded expression profile.

### 5.2 Enrichment Analysis of Angiogenic Factors Expression

We used the R package “limma” to identify AF-related differentially expressed genes (DEGs) (threshold: adj.P.Val<0.01 & |log (FC) |≥ 1) in 372 cancer and normal samples. Next, gene ontology (GO) enrichment analysis (*p*-value cutoff < 0.05) and KEGG pathway enrichment analysis (*p*-value cutoff < 0.05) of differentially expressed genes were performed using the R package “clusterProfiler”.

### 5.3 Univariate Cox Regression Analysis

Other data of cancer samples were further extracted, and a univariate Cox regression analysis of DEGs associated with overall survival was performed using the R packages “survival” and “survminer” with a threshold of *p* < 0.05. DEGs associated with prognosis were identified after screening.

### 5.4 Prognostic Risk Model Development Based on Lasso Regression

Lasso regression was performed using the R package “glmnet” for downscaling prognostic genes. We first screened lambda by cross-validation, and then selected the model with lambda. min. Next, the expression matrix of the selected genes for the model was extracted, and the risk score of each sample was calculated using the following formula:

$${\text{Riskscore}}_{\text{i}}=\sum_{\text{i}=1}^{\text{n}}{\text{exp}}_{\text{ji}}*\beta_{\text{j}}.$$

It represented the expression level of gene j in sample i, and represented the coefficient of gene j in the lasso regression model. All the samples were stratified into high- and low-risk groups according to the median-risk score.

### 5.5 Risk Model Assessment

Kaplan–Meier survival curves were plotted according to high- or low-risk groups. The ROC curves were drawn based on the predicted risk score of each sample.

### 5.6 Analysis of Angiogenic Factor Risk Scores According to Clinical Characteristics

The samples with AF risk scores were grouped according to clinical characteristics. We used the R package “ggplot2” to show the distribution of AF risk scores in each group and the R package “ggpubr” to illustrate the significant difference between groups.

### 5.7 Association Analysis of Angiogenic Factor Risk Score

We calculated tumor mutational burden, homologous recombination deficiency (HRD) (from technical support), tumor neoantigen burden (according to the literature The Immune Landscape of Cancer), chromosome instability (CIN) (according to the literature The Immune Landscape of Cancer), and stemness index (according to the literature Machine Learning Identifies Stemness Features Associated with Oncogenic Dedifferentiation) based on AF risk scores and performed association analyses.

### 5.8 Assessment of Immune Infiltration in the High- and Low-Risk Groups Using CIBERSORT

The proportion of 22 immune cells in the samples can be derived using the CIBERSORT algorithm based on the expression of certain genes. We assessed the difference in immune infiltration between the high- and low-risk groups by *t*-test with a significance threshold of *p* < 0.05.

### 5.9 Assessment of Immune Score, Stromal Score, and Tumor Purity Using ESTIMATE

We analyzed the differences in the immune score, stromal score, and tumor purity of AF in high- and low-risk groups using the R package “ESTIMATE” and assessed the differences in immune infiltration in the high- and low-risk groups by *t*-test with a threshold of *p* < 0.05.

### 5.10 Mutation Analysis in High and Low-Risk Groups

MAF files of NSCLC were downloaded from the GDC database, and we extracted the mutation information of AF from the somatic mutation profile. The mutation profile of AF in high- and low-risk groups was demonstrated with the help of the “oncoplot” function, using the R package “maftools”.

### 5.11 Copy Number Variation Analysis of High- and Low-Risk Groups

The copy number variation (CNV) data of LUSC and LUAD were downloaded from UCSC. The copy numbers of the high- and low-risk groups were extracted to generate files and plotted on the gadget using the “CNV distribution chart - bar graph section”.

### 5.12 Prediction of Response to Immunotherapy

The expression profiles of immune genes were extracted from the processed data of endometrial cancer samples, and the immune gene sets were obtained from the ImmPort database (https://www.immport.org/) and InnateDB database (https://www.innatedb.ca/). The expression profiles of the immune gene sets were subsequently normalized. The predicted TIDE scores of the samples were calculated using the TIDE online database. The distribution of TIDE scores in the high- and low-risk groups was illustrated with box plots using ggpubr, and the significance was tested by *t*-test.

### 5.13 Independent Prognostic Factor Analysis

To validate whether the risk score was an independent prognostic factor, univariate Cox regression analyses of the candidate prognostic factors using TCGA sample data were first performed, including risk score, age, tumor stage, and TNM stage. A multivariate Cox regression analysis was subsequently performed to assess the effect size of the risk score. We used the function cph in the R package “rms” to plot the nomograms and calibration plots for visualization.

### 5.14 Statistical Analysis

All statistical analyses were performed using R software version 4.0.3. *p* < 0.05 was set as the significance criterion.

## Data Availability

The original contributions presented in the study are included in the article/Supplementary Material, further inquiries can be directed to the corresponding author.

## Glossary

AFRS: angiogenic factors related risk score
AFs: angiogenic factors
CIN: chromosomal instability
CNV: copy number variation
DEGs: differentially expressed genes
EGFR: epidermal growth factor receptor
HRD: homologous recombination deficiency
LASSO: least absolute shrinkage and selection operator
WHO: World Health Organization
LSTm: large scale transition
NSCLC: non-small-cell lung cancer
SCLC: small cell lung cancer
SCNV: somatic copy number variations
SNPs: single nucleotide polymorphisms
TMB: tumor mutational burden
VEGF: vascular endothelial growth factor
VEGFR2: vascular endothelial growth factor receptor 2
