# Supplementary material for: Angiogenic Factor-Based Signature Predicts Prognosis and Immunotherapy Response in Non-Small-Cell Lung Cancer
Source: Front Genet. 2022 May 18;13:894024. doi: 10.3389/fgene.2022.894024 (PMC9158321; doi:10.3389/fgene.2022.894024)
Supplement: Supplementary file 1 [file DataSheet1.docx]

Supplementary Material

## Supplementary Figures


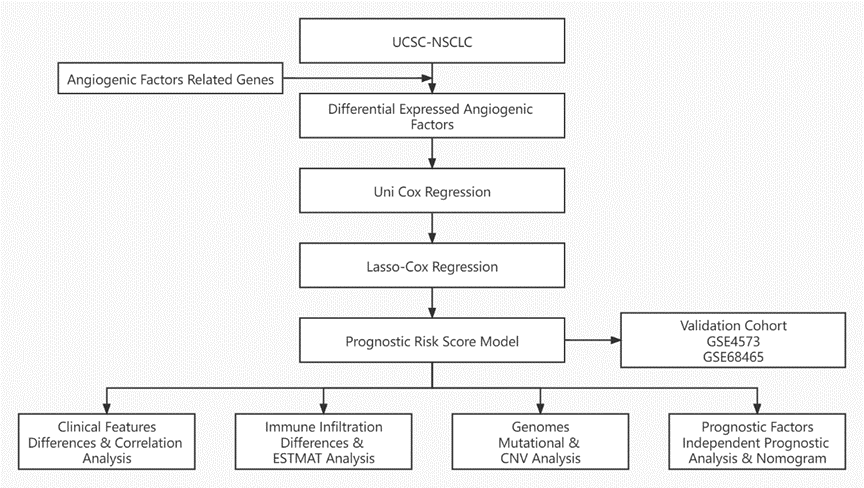


**Supplementary Figure 1.** Flow chart of the present study.


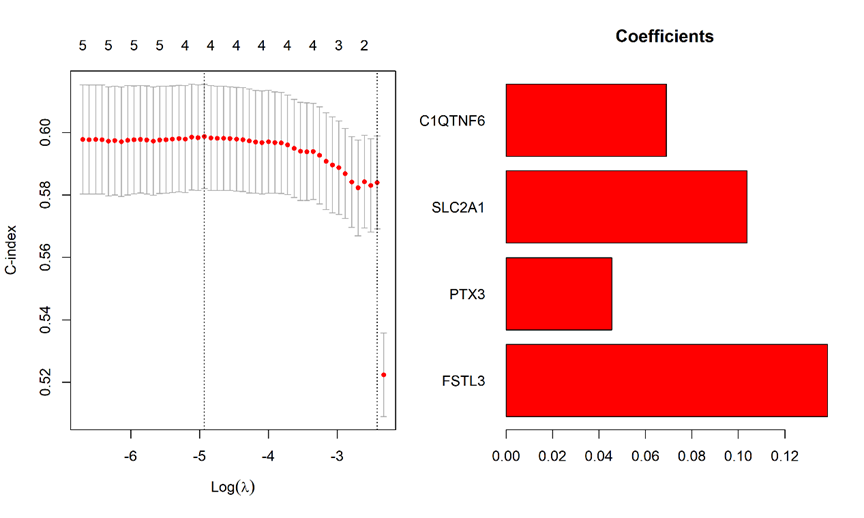


**Supplementary Figure 2.** Lasso regression.
